# The Present Status of Wound Treatment

**Published:** 1892-09

**Authors:** F. J. Thornbury

**Affiliations:** Buffalo, N. Y., Demonstrator of Bacteriology in the Medical and Dental Departments of the University of Buffalo; 610 Main Street


### THE PRESENT STATUS OF WOUND TREATMENT.

By F. J. THORNBURY, M. D., Buffalo, N. Y.,
Demonstrator of Bacteriology in the Medical and Dental Departments of the University of Buffalo.

The chief and most valuable lesson which the surgeon, educated now in the von Bergmann Clinic, has to learn, is the wide range of contrast existing between the present method of dealing with wounds and the manner formerly in vogue, comprising elaborate sponging and irrigation, both during the operation and at each subsequent change of dressing.

No other advance of modern surgery is more important than that which characterizes the evolution of antisepsis into asepsis, and the subsequent discontinuance of irrigation.

To Landerer belongs the credit of having first, in 1889, pointed out the superior advantages of the dry treatment of wounds. A trial of the method was at once begun in a few cases in the von Bergmann Clinic, and in a short time it found universal application by reason of the superiority in the method which became manifest. But for some time previous in the clinic, wounds had not been treated in the manner practised by most surgeons, who still inject into the wound chemical solutions under high pressure from an elevated reservoir connected by a rubber hose. On the contrary, the wounds were irrigated but slightly, and only with pure, sterilized water supplied through a small hand-irrigator.

The forcible irrigation of suppurating wounds does not simply wash away the purulent secretion, but, on the contrary, may tend to force the infectious material into the wound and rather disseminate than limit the infection.

When we look back to review the advent of antisepsis, we must conclude that it never had a thoroughly reliable basis, either experimental or as the result of surgical experience, but instead, the teaching was founded chiefly upon hypotheses and things taken for granted. The supposed necessity for irrigating wounds was accepted in a manner similar to that which characterized the popularity enjoyed by the spray for a short time.

The belief that a wound can be "disinfected" by means of antiseptic irrigation with the indications laid down for their application, have, like many other things, been inaugurated chiefly through custom, and transmitted from one clinician to another; thus finding a fixed place without due authenticity and reliability. Furthermore, the too slight consideration of the degrees of tolerability possessed by wounds, and, on the other hand, the actual disinfecting properties of antiseptic solutions, led of necessity very early to scepticism regarding the irrigation, *i. e.*, as to whether it really could accomplish all that was claimed for it, and all that it should.

Under no other circumstances are the conditions for the working of an antiseptic so unfavorable as by the method of irrigation as usually practised. Among the requirements of chemical disinfection are, first, the capability of permeating every infectious particle; second, the prevention of changes in the wound secretion, and finally, the assurance that these effects will be lasting. But the antiseptic fails to accomplish these requirements, because in fresh infected, and still more in old contaminated wounds, the septic germs — cocci and bacilli — are already enveloped in the blood clots and imbedded in the tissue particles and dried secretion or crusts, if they have not already invaded the interstitial connective tissue.

The ordinary antiseptics, especially sublimate and carbolic acid, have not the capability in diluted solutions of permeating these substances to come in contact with contained organisms.

The richly albuminous wound secretion enters into combination with the antiseptic during the irrigation, thereby reducing to a marked extent the effect of the agent, or destroying its action altogether.

Further, the short continuance of the application of the weak solutions would, of itself, preclude the probability of the resistant organisms being destroyed. In the one instance, therefore, we cannot be absolutely certain whether a disinfection of the wound is to be accomplished by means of the antiseptics. While, on the other hand, we can calculate with reasonable certainty on a greater or less amount of damage resulting. The latter pertains especially to the toxic and irritating properties of most of the solutions.

We must admit that the tissues themselves at the seat of application suffer more or less under the action of chemicals, and that the tissue cells are seriously damaged long before the much more resisting bacteria are destroyed. We are presented with evidence of this, practically, each time a fresh wound is irrigated with strong carbolic acid solution. The surface no longer appears bright and red, but assumes, rather, a pale whitish appearance, and becomes covered with small grayish, superficially located, corroded points.

Furthermore, the antiseptics cause especially active secretion, and interfere materially with the healing process. Frequently, as is well known, a general intoxication results, often of the severest form.

The one effect which the irrigation does possess is the removal of blood and purulent secretions, and the providing of an influence which limits the development of organisms in the wound, even though the solution used may not destroy the individual bacteria. But with reference to removal of blood or secretion, sponging away of the discharge with some absorbing material, such as hydrophile gauze, accomplishes the purpose better than the irrigation with even now irritating fluids as a physiological ($\frac{3}{4}$ per cent.) salt solution.

This method of drying the wounds with absorbing gauze finds application in the von Bergmann Clinic in all operations, and at the subsequent change of dressings, no irrigation being used. Only very exceptionally—in case of especially profuse discharge—is one of the foregoing solutions employed. Influences which limit the development of bacteria, or modify their ptomaïne action,— the so much favored sublimate, carbolic acid and other antiseptic solutions,—after all, as experience has shown, present the widest ranges of variation as to their efficacy.

A wound which is covered with green pus cannot be rendered absolutely free from liability to further development of the bacillus pyocyanins by the mere application of an antiseptic solution, excepting possibly by a very long continuance of the irrigation.

For limiting the development of bacteria and rendering the microbes inert, iodoform has long been substituted. In fresh wounds, especially, does it find preference of application. It is deodorizable with vanileine, is a mild agent, non-irritating and at the same time thoroughly antiseptic and capable of preventing the development of septic changes in the wound secretion.

Therefore, notwithstanding the repeated attacks which have been made upon this drug at various times, it still maintains its place at the head of the list of agents to be preferred in wound treatment.

But, in conclusion, we do not lay much stress upon antiseptics in general; little demand will be had for them if the rules of asepsis are properly observed.

610 Main Street.

